# AMPK-Targeted Effector Networks in Mycobacterial Infection

**DOI:** 10.3389/fmicb.2019.00520

**Published:** 2019-03-15

**Authors:** Eun-Kyeong Jo, Prashanta Silwal, Jae-Min Yuk

**Affiliations:** ^1^Department of Microbiology, School of Medicine, Chungnam National University, Daejeon, South Korea; ^2^Infection Control Convergence Research Center, School of Medicine, Chungnam National University, Daejeon, South Korea; ^3^Department of Medical Science, School of Medicine, Chungnam National University, Daejeon, South Korea; ^4^Department of Infection Biology, School of Medicine, Chungnam National University, Daejeon, South Korea

**Keywords:** AMPK, mycobacteria, macrophage, autophagy, mitochondria, immunometabolism

## Abstract

AMP-activated protein kinase (AMPK), a key metabolic regulator, plays an essential role in the maintenance of energy balance in response to stress. Tuberculosis (TB), primarily caused by the pathogen *Mycobacterium tuberculosis* (Mtb), remains one of the most important infectious diseases worldwide, characterized by both high incidence and mortality. Development of new preventive and therapeutic strategies against TB requires a profound understanding of the various host-pathogen interactions that occur during infection. Emerging data suggest that AMPK plays an essential regulatory role in host autophagy, mitochondrial biogenesis, metabolic reprogramming, fatty acid β-oxidation, and the control of pathologic inflammation in macrophages during Mtb infection. As described in this review, recent studies have begun to define the functional properties of AMPK modulators capable of restricting intracellular bacteria and promoting host defenses. Several host defense factors in the context of AMPK activation also participate in autophagic and non-autophagic pathways in a coordinated manner to enhance antimicrobial responses against Mtb infection. A better understanding of these AMPK-targeted effector networks offers significant potential for the development of novel therapeutics for human TB and other infectious diseases.

## Introduction

AMP-activated protein kinase (AMPK) is an essential metabolic sensor that responds to increases in the cellular adenosine monophosphate (AMP) to adenosine triphosphate (ATP) ratio (AMP:ATP) by activating catabolic pathways for energy production. In addition to metabolic stresses, the AMPK pathway is also activated in response to various stress-related signals including infection and inflammation. A wide range of infectious diseases have been shown to trigger AMPK signaling cascades, resulting in modulation of host responses that can either enhance pathogen survival or promote host defenses in a context-dependent manner (Grahame Hardie, 2016; Moreira et al., 2016; Prantner et al., 2017; Lee et al., 2018). Recent studies have revealed a diverse array of functions associated with the AMPK pathway, including regulation of host signaling, and participation in important defensive functions such as autophagy, mitochondrial biogenesis, metabolic reprogramming, and regulation of inflammation during infection (Prantner et al., 2017; Silwal et al., 2018). Each of these discoveries offers the potential of new therapeutic strategies capable of harnessing the AMPK pathway to combat diverse infectious diseases including human tuberculosis (TB).

*Mycobacterium tuberculosis* (Mtb) is the major causal pathogen of TB, which remains a global health problem, with a high prevalence of both multidrug-resistant and extensively drug-resistant TB (WHO, 2017). With approximately one third of the world’s population thought to be latently infected with Mtb, there remains an urgent need for new therapeutic developmental modalities. These advances, however, remain limited by an incomplete understanding of the host-pathogen interaction due in part to the complicated lifestyle of Mtb within host cells (Hmama et al., 2015; Dorhoi and Kaufmann, 2016). Mtb has a unique waxy coating on its cell wall comprised primarily of mycolic acids, a unique adaptation which enables survival within host cells (Daffe et al., 2014). In addition to its cell wall, Mtb has evolved multiple strategies to evade both innate and adaptive immune defenses, enabling both persistent infection and even active replication within the human host (Hmama et al., 2015), though the exact mechanisms underlying this survival remain poorly understood.

Upon Mtb infection, a variety of mycobacterial components including protein antigens and lipids trigger a series of innate inflammatory responses in host macrophages, though these pathogens can often resist these responses and escape from immune clearance (Dorhoi and Kaufmann, 2016). Despite this, excessive inflammatory responses by the host can often lead to unwanted pathological damage during infection (Cooper, 2009). Since Mtb can persist within the highly lipophilic replicative niche of macrophages for most of its life cycle, an intricate interconnection between bacterial and host cellular metabolism will ultimately determine the overall picture of host-pathogen interaction (Hmama et al., 2015). Autophagy, as a cell-autonomous quality control system, is a crucial process for maintaining homeostasis of the immune, inflammatory, and metabolic responses in host cells during infection (Deretic et al., 2015; Paik et al., 2018). Given the clear need for overcoming drug-resistant issues, many efforts are being made to develop host-targeted therapies to combat TB and other infections.

In this review, we summarize the current literature suggesting a role for AMPK as a central mediator regulating a diverse set of biological responses including autophagic, lysosomal, and metabolic pathways in the Mtb-infected host. In addition, we analyze the regulatory mechanisms underlying the beneficial antimicrobial effects mediated by AMPK signaling during Mtb infection. Finally, we discuss the advances and technical challenges surrounding the use of AMPK-targeting small molecules as novel therapeutic strategies for the treatment of TB.

## Overview of AMPK

AMP-activated protein kinase is a member of the serine/threonine (Ser/Thr) kinase family and is ubiquitously expressed in eukaryotic cells. AMPK monitors and senses the AMP/ADP relative to ATP to maintain an adequate energy supply by promoting catabolic pathways and/or decreasing anabolic pathways in response to stress conditions (Moreira et al., 2016). Maintaining proper ATP concentrations within cells is critical for cell survival, as dysregulation of energy homeostasis can lead to a wide range of pathologies including metabolic diseases, cardiovascular diseases, and cancer (Hardie, 2011a,b; Carling, 2017).

AMP-activated protein kinase exists as a heterotrimeric complex composed of a catalytic α subunit and two regulatory β and γ subunits (Hardie, 2011b; Hardie et al., 2016; Moreira et al., 2016). Furthermore, there are several isoforms for each subunit of AMPK (two for α and β subunits; three for γ subunits), which combine to form different AMPK complexes. As the catalytic subunit, the α subunit of AMPK complex is a main functional component and essential for AMPK activation through its phosphorylation of Thr172, whereas the γ subunit functions as a sensor of ADP levels and interacts with ADP (Novikova et al., 2015; Hardie et al., 2016; Moreira et al., 2016).

**FIGURE 1:**
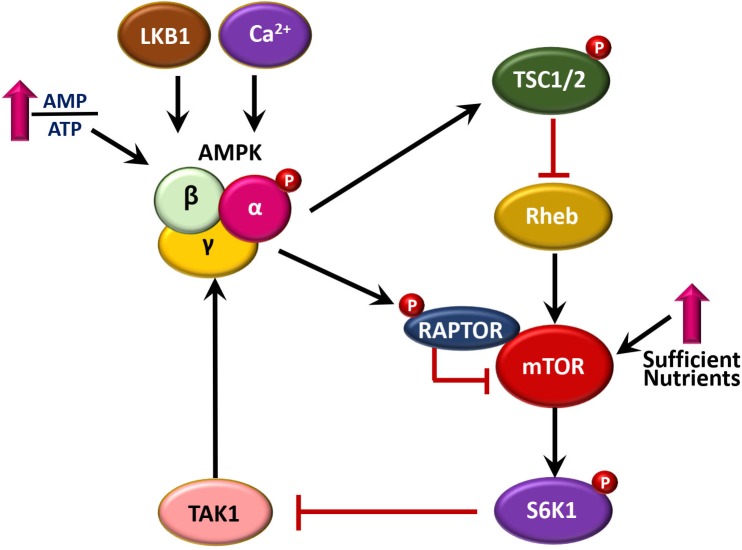
A brief summary of reciprocal regulation between AMPK and mTOR pathways. AMPK is activated by upstream signaling pathways, including LKB1, CaMKKII, and TAK1. AMPK pathway inhibits mTOR signaling, through at least two mechanisms, i.e., phosphorylation of TSC2 via Rheb, and direct phosphorylation of RAPTOR. In addition, mTOR-S6K1 signaling pathway inhibits AMPK activation through inactivation of TAK1.

AMP-activated protein kinase activation is mediated by several upstream signaling pathways, including the liver kinase B 1 (LKB1) tumor suppressor, as well as Ca^2+^/calmodulin-dependent kinase II (CaMKKII)-mediated phosphorylation of AMPK (Green et al., 2011; Marcelo et al., 2016). In addition, TGF-β-activated kinase-1 (TAK1) acts as an upstream kinase for AMPK (Xie et al., 2006; Inokuchi-Shimizu et al., 2014; Neumann, 2018; Silwal et al., 2018). Several lines of evidence showed a reciprocal regulation between AMPK and mTOR signaling pathways. AMPK phosphorylation leads to the inhibition of mammalian target of rapamycin (mTOR) through phosphorylation of tuberous sclerosis complex 2 (TSC2), which in turn affects both cell metabolism and growth (Corradetti et al., 2004; Inoki et al., 2012). Another mechanism by which AMPK inhibits mTOR activity is mediated through phosphorylation of RAPTOR (regulatory-associated protein of mTOR) (Gwinn et al., 2008). On the other hands, mTOR signaling suppresses the activation of AMPK through p70 S6 kinase 1 (S6K1)-mediated inhibition of TAK1 (Xu et al., 2017; Sun et al., 2018). Interestingly, AMPK and its upstream kinase LKB1, as well as mammalian target of rapamycin complex 1 (mTORC1), are primarily located at the surface of the lysosome where they coordinate various homeostatic signaling pathways such as autophagy (Korolchuk et al., 2011; Carroll and Dunlop, 2017). The elaborate mechanisms that delineate the reciprocal regulation between AMPK and mTOR pathways are shown in Figure 1.

Upon activation, AMPK functions as a signaling hub regulating a vast array of proteins, enzymes, metabolic pathways, and other signaling pathways (Hardie and Alessi, 2013; Moreira et al., 2016). Through its crosstalk with various signaling mediators, AMPK serves as a key modulator under diverse physiologic conditions, including cell growth, proliferation, autophagy, mitochondrial biogenesis, stress responses, and immune regulation (Hardie, 2011b; Shah et al., 2017). In addition, AMPK activation is critically required for controlling mitochondrial biogenesis and metabolic function to promote cell survival and keep homeostasis (Hardie, 2011b; Zhang et al., 2017). Under pathological condition, dysregulation of AMPK is associated with pathogenesis of various human diseases including inflammatory, metabolic, and neurodegenerative diseases (Ruderman et al., 2013; Peixoto et al., 2017; Szrejder and Piwkowska, 2019). Indeed, numerous pathogens can modulate AMPK activity to enhance or ameliorate host protective immune responses during infection (Moreira et al., 2016; Silwal et al., 2018). Moreover, AMPK activity is closely associated with immunometabolic regulation during a wide range of infections (Mahon and Hafner, 2015; Moreira et al., 2016). In addition to the roles described above, emerging evidence suggests that AMPK is involved in the activation of antimicrobial immunity and immunopathologic responses during mycobacterial infection through its interaction with other signaling pathways (Yang et al., 2014; Ong et al., 2015). These results, combined with other results which will be discussed in the later section of AMPK-targeting small molecules in this review, have shed the light on the development of potential therapeutics for host-directed therapy against TB and other infectious diseases. However, future studies are warranted to investigate the contribution of AMPK-targeted therapy *in vivo* and to delineate the mechanisms by which AMPK regulates innate immune responses during different stages of Mtb infection.

## AMPK: Autophagy-Inducing Kinase in Mycobacterial Infection

Autophagy is an intracellular pathway that is crucial for maintenance of homeostasis by triggering the degradation of long-lived proteins and unnecessary intracellular components in response to a variety of stresses (Mizushima et al., 2008; Choi et al., 2013). Numerous studies have demonstrated a role for autophagy in a variety of biological responses including the regulation of innate and adaptive immunity, and antimicrobial responses (Levine and Deretic, 2007; Mizushima et al., 2008). A full overview of the roles and mechanisms by which autophagy activates antimicrobial effector pathways and controls inflammation during mycobacterial infection has been reviewed elsewhere (Deretic, 2008, 2011; Deretic et al., 2009; Ni Cheallaigh et al., 2011; Liu et al., 2017; Paik et al., 2018). As an autophagy-activating kinase, AMPK is involved in the regulation of innate host defense against intracellular pathogens, including Mtb (Yang et al., 2014; Singh and Subbian, 2018). Here, we briefly discuss autophagy and mycobacterial infection, and the role of AMPK activation in the antimicrobial response to Mtb infection.

### AMPK Cross-Talk With Autophagy in Mycobacterial Infection

**FIGURE 2:**
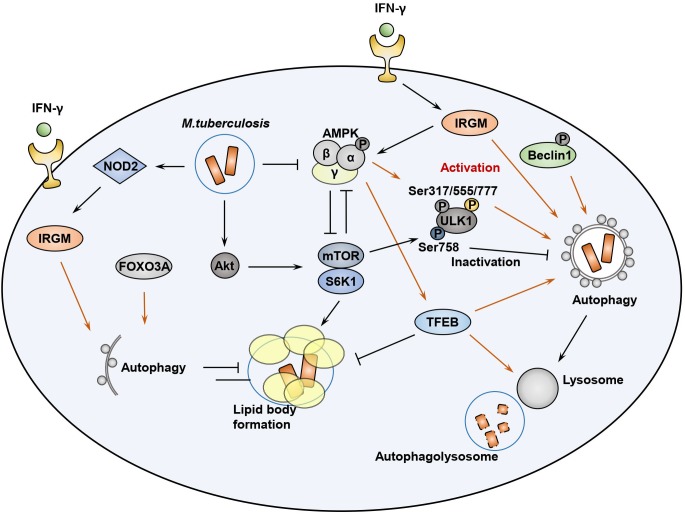
AMPK-mTOR signaling pathways during Mtb infection. During infection, Mtb can activate mTOR pathway to enhance lipid body formation and ULK1 inhibition. AMPK activation, which is induced by AMP/ATP ratio, LKB1, intracellular calcium influx, as well as IRGM, is required for autophagy activation through ULK1 phosphorylation at Ser317/555/777. Either adaptive (Th1 cytokine IFN-γ) or innate (NOD2) signaling can induce IRGM activation, which is required for autophagy activation. AMPK-mediated Beclin-1 phosphorylation also activates autophagy to enhance phagosomal maturation. AMPK and mTOR pathways reciprocally inhibit each other to regulate autophagy, metabolism, and inflammatory responses in host cells during Mtb infection. AMPK-mediated TFEB activation leads to lysosomal activation and fatty acid β-oxidation to suppress lipid body formation. Mtb-mediated Akt phosphorylation can inhibit Foxo3A activation, which is also required for autophagy activation in the host cells.

Initiation of macroautophagy is triggered by the UNC-51-like kinase 1/2 complex [ULK1 and ULK2, the mammalian orthologs of autophagy-related 1 (Atg1)] (Hara et al., 2008; Mercer et al., 2009) and serves as an important downstream effector. The resulting effector molecules are in turn regulated by the coordinated function of AMPK and mTORC1 (Figure 2) (Kim et al., 2011). Activated AMPK induces the phosphorylation of serine residues S317, S555, and S777 of ULK1, a mammalian autophagy-initiating kinase, which plays an important role in starvation-induced autophagy (Bach et al., 2011; Kim et al., 2011; Wong et al., 2013). Indeed, ULK1 has several phosphorylation sites relevant to this process, including mTORC1-mediated phosphorylation of ULK1, which disrupts the association between ULK1 and AMPK (Kim et al., 2011). Previous studies showed that Mtb infection of macrophages by the mTOR/70 kDa ribosomal S6 kinase 1 (S6K1) pathway serves as an inhibitor of host autophagy, as well as a driver of inflammatory responses (Yang et al., 2006, 2014). By contrast, induction of AMPK activation by AICAR resulted in phosphorylation of ULK1 at Ser317 and Ser777, thereby promoting host cell autophagy and antimicrobial responses in both *in vitro* macrophage assays and *in vivo* (Yang et al., 2014). These data suggest that Mtb infection drives activation of mTOR signaling, with the presumed benefits of nutrient support and immunologic shelter via lipid body formation in host macrophages. However, AMPK activation by autophagy-inducing agents may counteract the pathogen strategy and redirect intracellular defenses toward catabolism in the form of increased lysosomal activation. Given the well-established function of AMPK in autophagy activation and negative regulation of mTOR pathways, AMPK might represent a promising target of host-directed therapy against TB (Yao et al., 2016; Singh and Subbian, 2018).

The IFN-γ-inducible GTPase IRGM is an autophagy protein associated with Crohn’s disease and TB (Bekpen et al., 2010). Activation of this protein is essential for phagosomal maturation and elimination of Mtb in murine and human macrophages (MacMicking et al., 2003; Gutierrez et al., 2004; Singh et al., 2006; Singh et al., 2010). Nevertheless, how the IRGM GTPase exerts its innate effector function during intracellular infection remains poorly understood. One pathway that has been implicated in innate immunity involves IRGM-mediated stabilization of intracellular AMPK (total and activated forms of AMPK), which in turn helps preserve levels of ULK1, ATG14, and ATG16L1, thereby enabling formation of the autophagic machinery (Chauhan et al., 2015). In addition, AMPK promotes autophagy via Beclin-1 phosphorylation at Thr388, which is required for Beclin-1 interaction with phosphatidylinositol 3-kinase catalytic subunit type 3 (PIK3C3) and autophagy-related 14 (ATG14) (Zhang et al., 2016). Given the previous findings of NOD2-mediated IRGM ubiquitination and activation (Chauhan et al., 2015), this IRGM-AMPK signaling pathway may serve as a bridge connecting innate immune signaling with functional activation of the autophagic machinery in response to mycobacterial infection.

### AMPK and Lysosomal Function in Mycobacterial Infection

Transcription factor (TF) EB, a master gene for lysosomal biogenesis, is an essential upstream regulator of autophagy, serving as a transcriptional activator of numerous autophagy and lysosomal related genes (Settembre et al., 2011). Under basal conditions, TFEB is suppressed by the action of cathepsin B-mediated cleavage of the calcium channel MCOLN1 in lysosomes, thus controlling the number of autophagosomes and lysosomes (Man and Kanneganti, 2016). TFEB phosphorylation by the mTORC1 kinase retains TFEB in its inactivated state in the cytosol (Martina et al., 2012; Roczniak-Ferguson et al., 2012). However, recruitment of AMPK leads to the suppression of mTORC1 activity, thereby activating TFEB (Figure 2), which is then transported into the nucleus where it activates transcription of the autophagic and lysosomal machinery and can even activate ULK1 in cases of *Francisella novicida* infection (Qi et al., 2016). In addition, induction of phagocytosis via the Fc receptor can itself increase the expression of lysosomal proteins through nuclear translocation and activation of TFEB, serving as an essential step for lysosomal degradation of phagocytosed bacteria (Gray et al., 2016). A recent study showed that TRIM16, an interacting protein of galectin-3, played a protective role in controlling Mtb infection through cooperation with core autophagic machinery in autophagic responses, and simultaneously affected TFEB activation (Chauhan et al., 2016).

Previous studies showed a stronger activation of mTOR kinase phosphorylation, relative to AMPK, in macrophages following Mtb infection in a time-dependent manner (Yang et al., 2014). Data indicating that cytosolic Mtb co-localizes with the components of autophagic machinery, i.e., p62 and LC3, was evident in only 30% of total Mtb phagosomes (Watson et al., 2012), providing evidence for the idea that the majority of intracellular Mtb might stop the activation of xenophagy due to a downregulation of TFEB nuclear translocation by mTOR activation during infection. In addition, Mtb can induce diverse innate immune signaling responses which converge on the NF-κB pathway and manipulate the host protective immune responses (Schorey and Schlesinger, 2016; Stutz et al., 2018). Mtb infection enhances the levels of microRNA (miRNA)-33 and miR-33^∗^ via the NF-κB pathway to inhibit autophagy, lysosomal function, and fatty acid β-oxidation. In this setting, AMPK activation appears to be crucial for the induction of TFEB, which promoted lipid catabolism as well as xenophagy against Mtb (Ouimet et al., 2016).

As the role of AMPK as a key modulator of TFEB-mediated lysosomal activation becomes clearer, the mechanisms by which AMPK activates autolysosomal function in the context of host defense against mycobacterial infection have garnered greater attention. Our recent study highlighted the importance of TFEB activation in antimicrobial responses along with the dampening pathologic inflammation during Mtb infection (Kim Y.S. et al., 2017). Importantly, the activation of peroxisome proliferator-activated receptor (PPAR)-α, a nuclear receptor that interacts with AMPK during metabolism (Grabacka et al., 2016), was found to enhance lysosomal biogenesis and function via TFEB, as well as protective antimicrobial responses against Mtb (Kim Y.S. et al., 2017).

A recent study showed that lysosomal damage inhibits mTOR kinase activity by galectin-8 interaction with the mTOR apparatus (Jia et al., 2018). However, galectin-9 activates AMPK signaling in response to lysosomal injury, and the galectin-based signal-transduction system plays a critical role in the activation of autophagy and host defense against Mtb (Jia et al., 2018). Further investigations in the mechanisms by which the host integrates galectin-based signaling networks during persistent infection with Mtb will be important for the development of anti-mycobacterial therapies capable of providing protective immunity against infection.

The autophagosomal proteins soluble *N*-ethylmaleimide-sensitive factor attachment protein receptor (SNARE) and ATG14 are important in autophagosome–lysosome fusion (Itakura et al., 2012; Diao et al., 2015). Earlier studies showed that mannose-capped lipoarabinomannan of Mtb was an inhibitor of phagosomal acidification through a SNARE alteration, thus contributing to virulence during Mtb infection (Fratti et al., 2003). The autophagy protein Irgm1 has been shown to exert antimicrobial effects against mycobacterial infection through the recruitment and subsequent accumulation of PtdIns(3,4)P_2_ and PtdIns(3,4,5)P_3_ to phagosomal membranes. These proteins modify the ability of Irgm1 to enhance immunity to Mtb phagosomes via its interaction with SNARE effectors (Tiwari et al., 2009). Despite these observations, few studies have sought to examine the role of AMPK in the modulation of SNARE as part of the autophagic flux observed during mycobacterial infection. A future challenge will be to determine the distinct role of AMPK on the SNARE system and how it integrates complex signaling networks into lysosomal homeostasis during mycobacterial infection.

## Roles of AMPK in Immunometabolism During Mycobacterial Infection

In response to inflammation, innate immune cells undergo an immunometabolic shift from enhanced aerobic glycolysis in early phases to high oxidative metabolism in later stages, which is often the result of AMPK activation (O’Neill and Hardie, 2013; Kelly and O’Neill, 2015). In addition to autophagy regulation, AMPK plays a crucial regulator of mitochondrial function and metabolism, through sensing the intracellular energy levels in innate immune cells (Williams and O’Neill, 2018). Here we briefly discuss the immunometabolism and its control by AMPK signaling pathways in innate immune cells in terms of mycobacterial infection.

### Overview of Immunometabolism in Innate Immune Cells

Recent studies have highlighted on the metabolic reprogramming in various immune cells. In particular, macrophages and dendritic cells (DCs) are principal innate immune cells that participate in the initiation and resolution of inflammatory responses during infection (O’Neill and Hardie, 2013; Kelly and O’Neill, 2015; Williams and O’Neill, 2018). In M1 macrophages and DCs, the source of energy is mainly provided by upregulation of aerobic glycolysis, although less efficient in the ATP production. Utilizing glycolysis, M1 macrophages and DCs can respond to more rapidly than oxidative phosphorylation (OXPHOS), to meet the energy request for the generation of inflammatory responses and biosynthetic precursor molecules in the initial phase of innate immune responses (Galvan-Pena and O’Neill, 2014; Williams and O’Neill, 2018). M1 macrophages show the metabolic characteristics of broken Krebs cycle and decreased fatty acid oxidation (FAO) and OXPHOS (Galvan-Pena and O’Neill, 2014; Kelly and O’Neill, 2015; O’Neill and Pearce, 2016; Williams and O’Neill, 2018). In this process, the increase of certain metabolites such as succinate and citrate, byproducts of fragmented Krebs cycle, act as signaling molecules that modulate inflammatory pathways in M1 macrophages (Galvan-Pena and O’Neill, 2014; Kelly and O’Neill, 2015; O’Neill and Pearce, 2016; Williams and O’Neill, 2018). Recent studies also revealed that specific metabolic pathways and metabolites such as itaconate are crucial for anti-mycobacterial effects (Michelucci et al., 2013; Williams and O’Neill, 2018).

**FIGURE 3:**
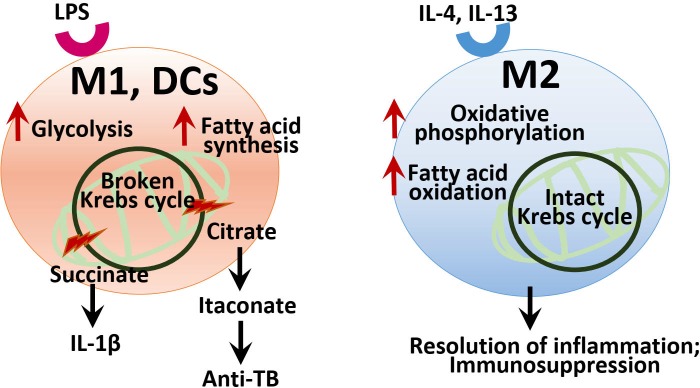
Summary of the immunometabolism in macrophages and DCs. In M1 and DCs, LPS stimulation leads to the upregulation of aerobic glycolysis, and altered TCA cycle with aberrant increase of several metabolic intermediates including succinate, citrate, and itaconate, which act as signaling and effector molecules in inflammatory responses and infection. In DCs, metabolic reprogramming results in the increased fatty acid synthesis through recharging NADPH and utilization of citrate. In M2 macrophages, the Krebs cycle and OXPHOS are intact to drive immunosuppression and the resolution of inflammation.

In DCs, TLR stimulation facilitates a metabolic shift to aerobic glycolysis that is linked to DC maturation and functional activation (Krawczyk et al., 2010; Cortese et al., 2014; Everts et al., 2014; O’Neill and Pearce, 2016). Importantly, the metabolic shift in DCs leads to the upregulation of fatty acid synthesis through pathways of recharging NADPH and utilization of citrate and isocitrate to efficiently accommodate cellular demand for protein synthesis during inflammation (Krawczyk et al., 2010; Cortese et al., 2014; Everts et al., 2014). Glycolysis is strongly upregulated in another inflammatory cells, Th17 cells, and functioned in the metabolic checkpoint regulation in T cell lineage differentiation (Shi et al., 2011; O’Neill et al., 2016). In murine M2 macrophages the Krebs cycle and OXPHOS are intact and functioning, and generate metabolic intermediates for protein glycosylation (Galvan-Pena and O’Neill, 2014; Jha et al., 2015; O’Neill and Pearce, 2016; Williams and O’Neill, 2018). It is now being recognized that immunoregulatory cells including M2 macrophages and regulatory T cells have a reliance on OXPHOS-dependent metabolism for their function (Williams and O’Neill, 2018). Taken together, Figure 3 summarizes a brief overview of immunometabolism in M1 and M2 macrophages, and DCs.

In innate immune cells, the pro-inflammatory signals inhibit AMPK activation in the initial stage of inflammation and negatively regulates the NF-κB signaling (Sag et al., 2008). Since AMPK senses the energy status in the cells undergoing inflammatory responses, its activation drives catabolic pathways such as FAO and OXPHOS, and simultaneously inhibits mTOR signaling and inflammatory pathways (O’Neill and Hardie, 2013; Kelly and O’Neill, 2015; Almeida et al., 2016; Williams and O’Neill, 2018). AMPK signaling, balanced with mTOR, appears to be a key immunometabolic regulator capable of influencing immune cell differentiation and is reinforced by autophagic function (Almeida et al., 2016; Riffelmacher et al., 2018). This review will focus on the role of AMPK in innate immune cell function during Mtb infection.

### Warburg Effect Mediated by HIF-1α During Mycobacterial Infection

**FIGURE 4:**
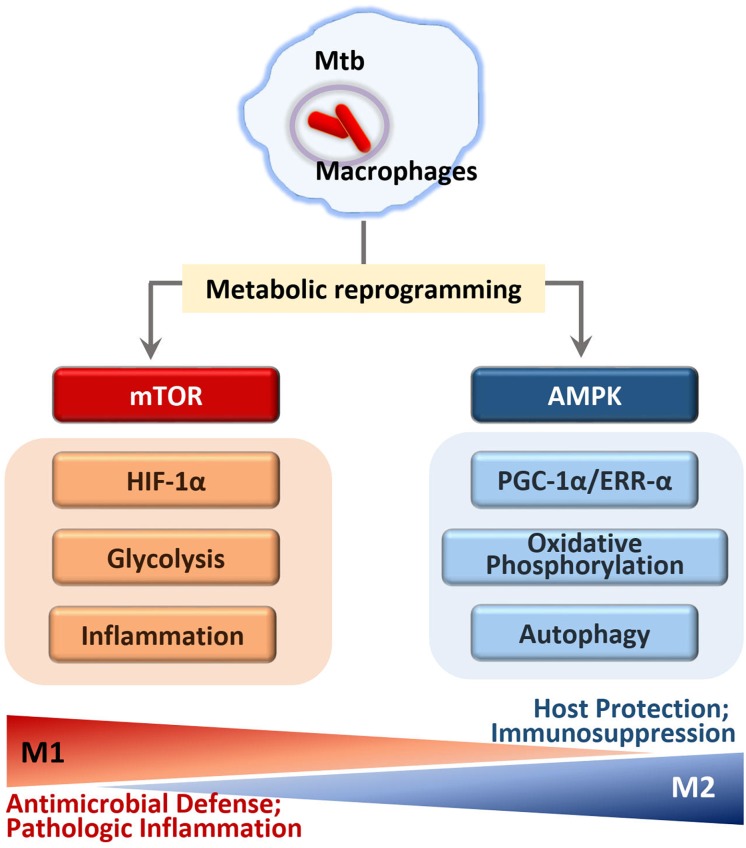
Regulation of immunometabolism in macrophages infected with Mtb. In early phase of Mtb infection, mTOR kinase activation leads to the immunometabolic shift to M1 macrophages that generate pro-inflammatory cytokine by activation of NF-κB and HIF1α signaling. M1-like macrophages increase glycolysis and inflammation through HIF1α and NF-κB signaling pathways. In later phase of infection, M2-like macrophages increase mitochondrial oxidative phosphorylation through AMPK activation. AMPK pathway enhances PGC1α and ERRα activity that is required for autophagy activation through transcriptional and translational regulation. Although the mTOR-HIF1α pathway is essential for initial control of Mtb growth, excessive induction of inflammation seems to be harmful to the host. Similarly, the prolonged activation of AMPK signaling to drive M2-like macrophages may result in the immunosuppression that is detrimental to eradicate intracellular mycobacteria.

Upon Mtb infection, the host immune responses are often confronted with the Warburg effect, a process through which immune cells efficiently meet cellular biosynthetic capacity to activate inflammatory responses and antimicrobial molecules (Shi et al., 2016; Riffelmacher et al., 2018). In this process, Mtb pathogenicity may be attributable to its perturbation of the Warburg effect, enabling escape from M1 macrophage polarization and Th1 protective immunity, to replicate and persist inside host cells (Figure 4) (Shi et al., 2016).

Mtb infection of macrophages or peripheral blood mononuclear cells induces strong activation of the mTOR/S6K1 and Akt signaling pathways to modulate host autophagy and metabolic processes (Yang et al., 2006, 2014; Lachmandas et al., 2016). The signaling pathways involving mTOR and Akt play a crucial role in the glycolytic shift in macrophages during infection (Matta and Kumar, 2016). As part of the bioenergetics shift to the Warburg effect, hypoxia-inducible factor (HIF)-1α, a downstream target of mTORC1 (Riffelmacher et al., 2018), promotes transcription of inflammatory genes as well as transcripts involved in aerobic glycolysis and *de novo* lipid synthesis in immune cells (Tannahill et al., 2013; Kelly and O’Neill, 2015; Riffelmacher et al., 2018). A recent study using an experimental TB model suggested a dual role of HIF-1α; i.e., a protective role during early stages of the disease, but detrimental in later phage of TB (Baay-Guzman et al., 2018). HIF-1α-mediated Warburg effect likely contributes to protective immune responses at the site of infection early in disease but may lead to injurious necrosis and pathology during a prolonged infection (Figure 4).

### AMPK-Mediated M1/M2 Regulation and Iron Homeostasis During Mtb Infection

Given the crucial function of AMPK in metabolic regulation, AMPK is often regarded as a master regulator of immunometabolism in immune cells, through orchestrating mitochondrial biogenesis, OXPHOS, and M2 macrophage differentiation (Figure 4) (O’Neill and Pearce, 2016; Griffiths et al., 2017). In M2 macrophages, the generation of proinflammatory cytokines decreased in response to Mtb infection, whereas the anti-inflammatory cytokines and growth factors are unaffected, maintaining normal function and tissue repair (Griffiths et al., 2017). Previous studies have largely found that M2 macrophages exhibit broad anti-inflammatory responses during Mtb infection, enabling a more permissive environment for intracellular mycobacteria growth (Sahu et al., 2017; Lugo-Villarino et al., 2018). By contrast, this type of immune response may also be advantageous to the host, protecting against potentially harmful immunopathology including extensive tissue damage (Pineros et al., 2017). Thus, AMPK may play as a checkpoint regulator of immunometabolism to tilt the balance to favor the increased host defense during Mtb infection.

A recent study has shown that ferritin heavy chain, a major factor regulating the preservation of iron in the host, is a critical factor in protective immunity, serving to ameliorate potentially pathologic inflammation caused by excessive mitochondrial activity and oxidative phosphorylation in host cells (Reddy et al., 2018). Given previous findings that AMPK is an important upstream regulator of ferritin heavy chain transcription (Iwasaki et al., 2013), further research into the role of AMPK in iron homeostasis and its effects on inflammation and immune responses against Mtb infection are warranted.

### AMPK-Induced Downstream Effectors That Coordinate Mitochondrial Function and Host Defense

In addition to these studies, previous work from our laboratory reported that AMPK activation resulted in increased mitochondrial biogenesis through peroxisome proliferator-activated receptor-γ coactivator (PGC)-1α, a downstream molecule of AMPK signaling (Yang et al., 2014). Importantly, PGC1α is critically involved in the antimicrobial immune defense and phagosomal maturation against Mtb (Yang et al., 2014). A more recent study revealed that estrogen-related receptor (ERR)-α, which is closely associated with PGC1α, was required for autophagy activation and host defense to Mtb infection (Kim et al., 2018). ERR-α works as an essential autophagy regulator by transcriptional and post-translational regulation of autophagy genes (Kim et al., 2018). Although ERR-α is an orphan nuclear receptor, for which the physiological ligands have not been known, ERR-α expression was upregulated by either AMPK or sirtuin 1 (SIRT1) activator (Kim et al., 2018). There is a mutual regulation between ERR-α and SIRT1 to enhance innate immune responses and ameliorate inflammation (Yuk et al., 2015; Kim et al., 2018). Treatment of Mtb-infected cells with SIRT1 activator resveratrol increased co-localization of Mtb phagosomes with lysosomes, suggesting an improved phagosomal maturation in macrophages (Kim et al., 2018). In addition, a recent study showed that SIRT1 is required for dampening immunopathogenesis of TB, suggesting a beneficial role for SIRT1 activation in host protection (Cheng et al., 2017). Moreover, AMPK activation is linked to mitochondrial biogenesis through activation of PGC1α and SIRT1 (Zhu et al., 2013; Marcinko and Steinberg, 2014; Wu et al., 2014; Tang, 2016). Nevertheless, more work is necessary to determine how PGC1α/ERRα/SIRT1 mediates anti-TB immunity, and whether AMPK-induced mitochondrial biogenesis itself serves to limit intracellular mycobacterial survival.

## Small Molecules That Modulate AMPK to Regulate Host Defense against Mtb Infection

Given the opposing roles of AMPK and mTOR, significant attention has been given to small molecules and other compounds capable of activating AMPK or inhibiting mTOR as a potential therapeutic strategy for the host-directed therapy against TB (Floto et al., 2007; Schiebler et al., 2015; Singh and Subbian, 2018). Earlier studies suggest that pharmacologic inhibition of mTOR by rapamycin promotes phagosomal colocalization with autophagosomes and restriction of intracellular mycobacterial growth in macrophages (Gutierrez et al., 2004). Rapamycin is well-known for immunosuppressive activity to be useful for prevention of organ transplantation rejection (Pascual et al., 2016). Several mTOR inhibitors including everolimus are being used in the treatment of various types of cancers due to the inhibitory function in cell proliferation (Pohanka, 2006; Koh et al., 2013). Although there might be a possibility of mTOR inhibitors as candidates of host-directed therapy against multi-drug-resistance (MDR)- or extensively drug-resistance (XDR)-TB, the impact for mTOR inhibitor-based adjunctive therapy in the context of TB remains to be determined, because of serious side effects such as hyperglycemia and immunosuppression (Singh and Subbian, 2018).

Targeting AMPK in mycobacterial infection appears to promote anti-mycobacterial effects and inhibit much of the exaggerated inflammatory responses associated with disease pathology. Indeed, numerous autophagy-activating agents appear to work by triggering the AMPK pathway, making this protein a useful target for modulating the antimicrobial response to Mtb (Hoyer-Hansen and Jaattela, 2007; Fabri et al., 2011). In human monocytes/macrophages, vitamin D induces antimicrobial effects through autophagy activation via cathelicidin expression (Yuk et al., 2009). Four-phenylbutyrate and vitamin D-induced autophagy was dependent on the endogenous antimicrobial protein LL-37, of which LL-37-mediated autophagy required activation of the AMPK pathway (Rekha et al., 2015). In addition, activation of functional vitamin D receptor signaling by the mycobacterial antigen LpqH was shown to be dependent on the AMPK-mediated p38 MAPK pathway to produce cathelicidin LL-37 for antimicrobial responses against Mtb infection (Shin et al., 2010). Furthermore, AMPK activator AICAR showed beneficial effects through the enhancement of bacterial colocalization with autophagosomes to enhance phagosomal maturation and antimicrobial responses against Mtb infection (Yang et al., 2014). AICAR treatment of macrophages led to an induction of DRAM2, an autophagy protein targeted by miR144^∗^ in human monocytes/macrophages, to enhance host defense to Mtb (Kim J.K. et al., 2017). A recent study also showed that ohmyungsamycins, a class of newly identified cyclic peptides, activated autophagy and antimicrobial effects, and attenuated inflammatory responses against Mtb through AMPK pathway activation (Kim T.S. et al., 2017).

Notably, metformin, the well-known antidiabetic drug and AMPK activator, was shown to be beneficial for host protection, even in cases of drug-resistant Mtb (Singhal et al., 2014). As a treatment for Mtb, AMPK activation is required for metformin-mediated reduction of Mtb growth in macrophages. In addition, metformin treatment was shown to ameliorate chronic inflammation and lung pathology and improved bacterial control in a mouse model of infection. In TB patients, metformin therapy was associated with improvements in several clinical parameters, including disease severity (Singhal et al., 2014). The dual role for metformin in diabetes and Mtb infection is important, as diabetes itself is an independent risk factor for active TB, and is associated with TB severity (Jeon and Murray, 2008); TB may lead to glucose intolerance, with TB patients often experiencing difficulty in glycemic control (Yorke et al., 2017). While still unproven, targeting AMPK represents an exciting new treatment option for TB combined with diabetes, with strong efficacy in animal models of TB driven by its support of healthy mitochondria, reduced inflammation, and regulation of autophagy and apoptosis (Madhavi et al., 2018). Studies involving the functions of AMPK in mycobacterial infection is summarized in Table 1. More comprehensive studies are warranted to explore the prospect of targeting AMPK in patients with both diabetes and TB.

**Table 1:** Summary of studies employing the functions of AMPK in mycobacterial infection.

Effectors targeting AMPK	Mycobacterial strains	Observed autophagy/immunometabolism outcome	Observed antimycobacterial effects	Type of cells	Type of animal, human subjects	Reference
AICAR	• M. tuberculosis • M. marinum • M. bovis BCG	• ↑ AMPK activation and antibacterial autophagy against *M. tuberculosis* in AICAR-treated cells • ↑ Oxidative phosphorylation, mitochondrial ATP production, and biogenesis • ↑ Transcriptional activation of multiple autophagy-related genes	• ↓ Intracellular survival in *M. tuberculosis*-infected cells • ↓ Mortality and bacterial burden in *M. marinum*-infected Drosophila melanogaster • ↓ CFU of M. bovis BCG in the spleen of Atg7fl/fl LysM-Cre- mice, but not Atg7fl/fl LysM-Cre+ mice (about 1 log reduction)	• BMDM • Raw 264.7 • THP-1	• Atg7fl/fl LysM-Cre+ mice • Spargel mutant Drosophila	Yang et al., 2014
LpqH	• *M. tuberculosis* H37Rv	• ↑ Antibacterial autophagy in LpqH-treated cells • ↑ C/EBP-β-dependent induction of Cyp27b1 hydroxylase • ↑ Cathelicidin expression • ↑ Intracellular Ca^2+^/AMPK/p38 MAPK signaling pathway	• ↓Survival rate of intracellular Mtb by 35-42% in LpqH-treated human monocytes	• Monocytes • Human MDM • THP-1		Shin et al., 2010
Phenylbutyrate	• *M. tuberculosis* H37Rv	• ↑ LL-37 expression and autophagy activation through Intracellular Ca^2+^, AMPK, and PtdIns3K signaling pathway • ↑ Expression of the autophagy-related genes BECN1 and ATG5 • ↑ Colocalization of LC3-II and LL-37 in the autophagosome	• ↓ Intracellular Mtb growth in phenylbutyrate-treated human MDM (40–60% growth inhibition, *P* = 0.002)	• Monocytes • Human MDM • THP-1		Rekha et al., 2015
miR-33 miR-33^∗^	• *M. tuberculosis* H37Rv	• ↑ lipid body formation in Mtb-infected macrophages • ↓ fatty acid oxidation in Mtb-infected macrophages • ↓ induction of autophagy via the regulation of autophagy gene expression • ↓ Mtb killing *in vitro* BMDM and *in vivo* mice model • ↓ expression of AMPKα and downstream transcription factors	• ↑ Dead or metabolically inactive Mtb by inhibition of miR-33 and miR-33^∗^ in peritoneal macrophage and BMDM (the dual Immunofluorescence viability assay and CFU assay) • ↓ Bacterial counts in the lung of hematopoietic miR-33 deficient mice on day 53 post-infection	• BMDM • Alveolar Macrophages • THP-1 • HEK293 cells	• Prkab1-/- mice • Atg16fl/fl LysM-Cre+ mice • Mir33-/- mice	Ouimet et al., 2016
miR-144	• *M. tuberculosis* H37Rv • Mtb-ERFP	• ↓ induction of autophagy via post-transcriptional regulation of DRAM2 • ↑ Mtb infection, ↓ activators of the AMPK pathway such as AICAR and vitamin D3	• Intracellular Mtb growth: Human MDMs transfected with miR-144 inhibitor (↓) or miR-144 mimic (↓)	• Human PBMC • Human MDM • THP-1 • HEK293 cells	• Lung and lymph node tissue from TB patients	Kim J.K. et al., 2017
Ohmyungsamycin A and B	• *M. tuberculosis* H37Rv • Mtb-ERFP • M. marinum	• ↑ killing of mycobacteria *in vitro* macrophages and *in vivo D. melanogaster* infection model • ↑ Antibacterial autophagy and Mtb phagosome maturation via AMPK signaling pathway
		• ↓ Mtb-induced inflammatory responses via AMPK signaling	• Evaluation of antibacterial properties against Mtb: MIC50 values for OMS-A and –B were 57 nM and 117 nM, respectively (REMA method), ↓ intracellular Mtb growth in BMDMs (CFU assay) • ↓ Mortality and bacterial burden in *M. marinum*-infected *Drosophila melanogaster*	• BMDM • Human MDM	• *Atg7*^fl^/^fl^LysM-Cre+ mice • *Atg7* mutant *Drosophila*	Kim T.S. et al., 2017
Galectin 8 and 9	• *M. tuberculosis* Erdman	• ↓ mTOR signaling by Galectin 8 via interaction the Ragulator-Rag SLC38A9 system • ↑ AMPK signaling by Galectin 9 through the engagement of TAK1 • ↑ Autophagy and antimicrobial host defense against Mtb	• ↑ Susceptibility in Mtb Erdman-infected Gal8 KO mice, as compared to WT littermates	• Human MDM • BMDM • THP-1 • HEK293 cells • HeLa cells	• *Atg5*^fl^/^fl^ LysM-Cre+ mice • Gal8 *Atg5*^fl^/^fl^ LysM-Cre+ mice	Jia et al., 2018
Metformin	• *M. tuberculosis* H37Rv • *M. bovis* BCG	• ↓ Intracellular growth of Mtb by mitochondrial ROS generation • ↑ Efficacy of anti-TB drugs in Mtb-infected mice • ↓ Tissue pathology, ↑Immune responses in Mtb-infected mice • ↓ Inflammatory responses in Mtb-infected lung and spleen cells • ↑ Clinical outcome in TB patients with type 2 diabetes mellitus	• ↓ Intracellular Mtb growth in Metformin-treated human MDM in a dose and time-dependent manner (CFU assay) • ↓ Intracellular survival of MDR strains of Mtb in THP-1 cells • ↓ Bacterial load in the lungs and spleens of Mtb-infected mice with metformin monotherapy (250 or 500 mg/kg) or combined therapy (isoniazid of ethionamide)	• Human MDM • THP-1 • Murine spleen and lung cells • Murine BMDC	• C57BL/6 mice • Evaluation of clinical outcome in patients from TB and diabetic cohorts	Singhal et al., 2014


*AICAR, AMP-mimetic 5-aminoimidazole-4-carboxamide-1- β-D-ribofuranoside; BMDM, bone marrow-derived macrophages; Cyp27b1, 25-hydroxycholecalciferol-1 α-hydroxylase; C/EBP, CCAAT/enhancer-binding protein; MAPK, mitogen-activated protein kinase; MDM, monocyte-derived macrophage; PtdIns3K, phosphatidylinositol 3-kinase; BECN1, Beclin 1, autophagy related; ATG, autophagy related gene; ERFP, enhanced red fluorescent protein; DRAM2, DNA damage-regulated autophagy modulator 2; PBMC, peripheral blood mononuclear cell; BMDC, Bone marrow-derived dendritic cells; MDR, multi-drug-resistance; ↑, increase; ↓, decrease.*

Finally, it should be noted that AMPK has been shown to drive immunopathology through the production of neutrophil MMP-8 (Ong et al., 2015). In addition, there is evidence of potential side effects associated with AMPK modulators (Choi et al., 2008; Wang and Hoyte, 2018). Studies employing the role of AMPK in mycobacterial infection are summarized in Table 1. Future development of new therapeutics targeting AMPK should consider the crosstalk of this kinase with other essential signaling pathways to achieve acceptable efficacy and safety while minimizing unwanted side effects during TB treatment.

## Conclusion

Since its discovery as a pivotal regulator of intracellular bioenergetics, AMPK has emerged as a central coordinator of a variety of biological responses, including autophagy, inflammation, and metabolic reprogramming during mycobacterial infection (Shi et al., 2016; Riffelmacher et al., 2018). The functions of AMPK are diametrically opposed by those of mTOR, and the balance of the activation of both kinases is reflected in the signaling pathways that converge on host defense in host cells against Mtb infection (Yang et al., 2014). AMPK promotes the induction and maturation of autophagy in response to infectious stresses and activates the transcriptional factor TFEB, which targets specific signaling modules for lysosomal function and fatty acid β-oxidation (Ouimet et al., 2016). AMPK-mediated autophagy activation provides a cell-autonomous defense framework, which is required for enhancement of phagosomal acidification and restriction of intracellular parasites. In addition, AMPK signaling is tightly coupled to the attenuation of pathologic inflammation, which can be harmful to the host during prolonged infection with Mtb.

Understanding how AMPK-mTOR signaling functions in response to infectious threats, and how these pathways orchestrate immunometabolic responses is essential for proper control of infectious diseases. Although AMPK activation appears to be involved in the host protection to Mtb infection through downstream regulators including PGC-1α, ERRα, SIRT1, and PPARα, little is known about the full picture of AMPK-induced effector pathways. Many questions remain unanswered, including the mechanisms by which host defenses and pathology are controlled over the course of infection, and the nature of key players that participate in the control of metabolic shifts during TB. While AMPK is well established as a central regulator of innate effector networks, a better understanding of how AMPK orchestrates this complicated host-pathogen response will be necessary for the development of new therapeutic approaches for TB. Thus, a challenge for the future studies involves coordinating a dedicated mechanism of AMPK-targeted effector pathways that integrate the antibacterial autophagy, controlling pathologic inflammation, mitochondrial biogenesis, and immunometabolism during infection.

## Author Contributions

E-KJ and J-MY contributed to conception and design of the study. E-KJ wrote the first draft of the manuscript. E-KJ, PS, and J-MY wrote sections of the manuscript. All authors contributed to manuscript revision, read and approved the submitted version.

## Conflict of Interest Statement

The authors declare that the research was conducted in the absence of any commercial or financial relationships that could be construed as a potential conflict of interest.

## References

[B1] Almeida L. Lochner M. Berod L. Sparwasser T. (2016). Metabolic pathways in T cell activation and lineage differentiation. *Semin. Immunol.* 28 514–524. 10.1016/j.smim.2016.10.009 27825556

[B2] Baay-Guzman G. J. Duran-Padilla M. A. Rangel-Santiago J. Tirado-Rodriguez B. Antonio-Andres G. Barrios-Payan J. (2018). Dual role of hypoxia-inducible factor 1 alpha in experimental pulmonary tuberculosis: its implication as a new therapeutic target. *Future Microbiol.* 13 785–798. 10.2217/fmb-2017-0168 29848058

[B3] Bach M. Larance M. James D. E. Ramm G. (2011). The serine/threonine kinase ULK1 is a target of multiple phosphorylation events. *Biochem. J.* 440 283–291. 10.1042/BJ20101894 21819378

[B4] Bekpen C. Xavier R. J. Eichler E. E. (2010). Human IRGM gene “to be or not to be”. *Semin. Immunopathol.* 32 437–444. 10.1007/s00281-010-0224-x 20737271

[B5] Carling D. (2017). AMPK signalling in health and disease. *Curr. Opin. Cell Biol.* 45 31–37. 10.1016/j.ceb.2017.01.005 28232179

[B6] Carroll B. Dunlop E. A. (2017). The lysosome: a crucial hub for AMPK and mTORC1 signalling. *Biochem. J.* 474 1453–1466. 10.1042/BCJ20160780 28408430

[B7] Chauhan S. Kumar S. Jain A. Ponpuak M. Mudd M. H. Kimura T. (2016). TRIMs and galectins globally cooperate and TRIM16 and galectin-3 co-direct autophagy in endomembrane damage homeostasis. *Dev. Cell* 39 13–27. 10.1016/j.devcel.2016.08.003 27693506 PMC5104201

[B8] Chauhan S. Mandell M. A. Deretic V. (2015). IRGM governs the core autophagy machinery to conduct antimicrobial defense. *Mol. Cell* 58 507–521. 10.1016/j.molcel.2015.03.020 25891078 PMC4427528

[B9] Cheng C. Y. Gutierrez N. M. Marzuki M. B. Lu X. Foreman T. W. Paleja B. (2017). Host sirtuin 1 regulates mycobacterial immunopathogenesis and represents a therapeutic target against tuberculosis. *Sci. Immunol.* 2:eaaj1789. 10.1126/sciimmunol.aaj1789 28707004 PMC5505666

[B10] Choi A. M. Ryter S. W. Levine B. (2013). Autophagy in human health and disease. *N. Engl. J. Med.* 368 1845–1846. 10.1056/NEJMc1303158 23656658

[B11] Choi K. Mollapour E. Choi J. H. Shears S. B. (2008). Cellular energetic status supervises the synthesis of bis-diphosphoinositol tetrakisphosphate independently of AMP-activated protein kinase. *Mol. Pharmacol.* 74 527–536. 10.1124/mol.107.044628 18460607 PMC2632961

[B12] Cooper A. M. (2009). Cell-mediated immune responses in tuberculosis. *Annu. Rev. Immunol.* 27 393–422. 10.1146/annurev.immunol.021908.132703 19302046 PMC4298253

[B13] Corradetti M. N. Inoki K. Bardeesy N. DePinho R. A. Guan K. L. (2004). Regulation of the TSC pathway by LKB1: evidence of a molecular link between tuberous sclerosis complex and Peutz-jeghers syndrome. *Genes Dev.* 18 1533–1538. 10.1101/gad.1199104 15231735 PMC443516

[B14] Cortese M. Sinclair C. Pulendran B. (2014). Translating glycolytic metabolism to innate immunity in dendritic cells. *Cell Metab.* 19 737–739. 10.1016/j.cmet.2014.04.012 24807219 PMC4050200

[B15] Daffe M. Crick D. C. Jackson M. (2014). Genetics of capsular polysaccharides and cell envelope (glyco)lipids. *Microbiol. Spectr.* 2:14. 10.1128/microbiolspec.MGM2-0021-2013 25485178 PMC4255156

[B16] Deretic V. (2008). Autophagy, an immunologic magic bullet: *Mycobacterium tuberculosis* phagosome maturation block and how to bypass it. *Future Microbiol.* 3 517–524. 10.2217/17460913.3.5.517 18811236 PMC3225291

[B17] Deretic V. (2011). Autophagy in immunity and cell-autonomous defense against intracellular microbes. *Immunol. Rev.* 240 92–104. 10.1111/j.1600-065X.2010.00995.x 21349088 PMC3057454

[B18] Deretic V. Delgado M. Vergne I. Master S. De Haro S. Ponpuak M. (2009). Autophagy in immunity against *Mycobacterium tuberculosis*: a model system to dissect immunological roles of autophagy. *Curr. Top. Microbiol. Immunol.* 335 169–188. 10.1007/978-3-642-00302-8-8 19802565 PMC2788935

[B19] Deretic V. Kimura T. Timmins G. Moseley P. Chauhan S. Mandell M. (2015). Immunologic manifestations of autophagy. *J. Clin. Invest.* 125 75–84. 10.1172/JCI73945 25654553 PMC4350422

[B20] Diao J. Liu R. Rong Y. Zhao M. Zhang J. Lai Y. (2015). ATG14 promotes membrane tethering and fusion of autophagosomes to endolysosomes. *Nature* 520 563–566. 10.1038/nature14147 25686604 PMC4442024

[B21] Dorhoi A. Kaufmann S. H. (2016). Pathology and immune reactivity: understanding multidimensionality in pulmonary tuberculosis. *Semin. Immunopathol.* 38 153–166. 10.1007/s00281-015-0531-3 26438324

[B22] Everts B. Amiel E. Huang S. C. Smith A. M. Chang C. H. Lam W. Y. (2014). TLR-driven early glycolytic reprogramming via the kinases TBK1-IKKvarepsilon supports the anabolic demands of dendritic cell activation. *Nat. Immunol.* 15 323–332. 10.1038/ni.2833 24562310 PMC4358322

[B23] Fabri M. Realegeno S. E. Jo E. K. Modlin R. L. (2011). Role of autophagy in the host response to microbial infection and potential for therapy. *Curr. Opin. Immunol.* 23 65–70. 10.1016/j.coi.2010.10.010 21071195 PMC3042547

[B24] Floto R. A. Sarkar S. Perlstein E. O. Kampmann B. Schreiber S. L. Rubinsztein D. C. (2007). Small molecule enhancers of rapamycin-induced TOR inhibition promote autophagy, reduce toxicity in huntington’s disease models and enhance killing of mycobacteria by macrophages. *Autophagy* 3 620–622. 10.4161/auto.4898 17786022

[B25] Fratti R. A. Chua J. Vergne I. Deretic V. (2003). *Mycobacterium tuberculosis* glycosylated phosphatidylinositol causes phagosome maturation arrest. *Proc. Natl. Acad. Sci. U.S.A.* 100 5437–5442. 10.1073/pnas.0737613100 12702770 PMC154363

[B26] Galvan-Pena S. O’Neill L. A. (2014). Metabolic reprograming in macrophage polarization. *Front. Immunol.* 5:420. 10.3389/fimmu.2014.00420 25228902 PMC4151090

[B27] Grabacka M. Pierzchalska M. Dean M. Reiss K. (2016). Regulation of ketone body metabolism and the role of PPARalpha. *Int. J. Mol. Sci.* 17:E2093. 10.3390/ijms17122093 27983603 PMC5187893

[B28] Grahame Hardie D. (2016). Regulation of AMP-activated protein kinase by natural and synthetic activators. *Acta Pharm. Sin. B* 6 1–19. 10.1016/j.apsb.2015.06.002 26904394 PMC4724661

[B29] Gray M. A. Choy C. H. Dayam R. M. Ospina-Escobar E. Somerville A. Xiao X. (2016). Phagocytosis enhances lysosomal and bactericidal properties by activating the transcription factor TFEB. *Curr. Biol.* 26 1955–1964. 10.1016/j.cub.2016.05.070 27397893 PMC5453720

[B30] Green A. S. Chapuis N. Lacombe C. Mayeux P. Bouscary D. Tamburini J. (2011). LKB1/AMPK/mTOR signaling pathway in hematological malignancies: from metabolism to cancer cell biology. *Cell Cycle* 10 2115–2120. 10.4161/cc.10.13.16244 21572254

[B31] Griffiths H. R. Gao D. Pararasa C. (2017). Redox regulation in metabolic programming and inflammation. *Redox Biol.* 12 50–57. 10.1016/j.redox.2017.01.023 28212523 PMC5312548

[B32] Gutierrez M. G. Master S. S. Singh S. B. Taylor G. A. Colombo M. I. Deretic V. (2004). Autophagy is a defense mechanism inhibiting BCG and *Mycobacterium tuberculosis* survival in infected macrophages. *Cell* 119 753–766. 10.1016/j.cell.2004.11.038 15607973

[B33] Gwinn D. M. Shackelford D. B. Egan D. F. Mihaylova M. M. Mery A. Vasquez D. S. (2008). AMPK phosphorylation of raptor mediates a metabolic checkpoint. *Mol. Cell* 30 214–226. 10.1016/j.molcel.2008.03.003 18439900 PMC2674027

[B34] Hara T. Takamura A. Kishi C. Iemura S. Natsume T. Guan J. L. (2008). FIP200, a ULK-interacting protein, is required for autophagosome formation in mammalian cells. *J. Cell Biol.* 181 497–510. 10.1083/jcb.200712064 18443221 PMC2364687

[B35] Hardie D. G. (2011a). AMP-activated protein kinase: a cellular energy sensor with a key role in metabolic disorders and in cancer. *Biochem. Soc. Trans.* 39 1–13. 10.1042/BST0390001 21265739

[B36] Hardie D. G. (2011b). AMP-activated protein kinase: an energy sensor that regulates all aspects of cell function. *Genes Dev.* 25 1895–1908. 10.1101/gad.17420111 21937710 PMC3185962

[B37] Hardie D. G. Alessi D. R. (2013). LKB1 and AMPK and the cancer-metabolism link - ten years after. *BMC Biol.* 11:36. 10.1186/1741-7007-11-36 23587167 PMC3626889

[B38] Hardie D. G. Schaffer B. E. Brunet A. (2016). AMPK: an energy-sensing pathway with multiple inputs and outputs. *Trends Cell Biol.* 26 190–201. 10.1016/j.tcb.2015.10.013 26616193 PMC5881568

[B39] Hmama Z. Pena-Diaz S. Joseph S. Av-Gay Y. (2015). Immunoevasion and immunosuppression of the macrophage by *Mycobacterium tuberculosis*. *Immunol. Rev.* 264 220–232. 10.1111/imr.12268 25703562

[B40] Hoyer-Hansen M. Jaattela M. (2007). AMP-activated protein kinase: a universal regulator of autophagy? *Autophagy* 3 381–383. 17457036 10.4161/auto.4240

[B41] Inoki K. Kim J. Guan K. L. (2012). AMPK and mTOR in cellular energy homeostasis and drug targets. *Annu. Rev. Pharmacol. Toxicol.* 52 381–400. 10.1146/annurev-pharmtox-010611-134537 22017684

[B42] Inokuchi-Shimizu S. Park E. J. Roh Y. S. Yang L. Zhang B. Song J. (2014). TAK1-mediated autophagy and fatty acid oxidation prevent hepatosteatosis and tumorigenesis. *J. Clin. Invest.* 124 3566–3578. 10.1172/JCI74068 24983318 PMC4109552

[B43] Itakura E. Kishi-Itakura C. Mizushima N. (2012). The hairpin-type tail-anchored SNARE syntaxin 17 targets to autophagosomes for fusion with endosomes/lysosomes. *Cell* 151 1256–1269. 10.1016/j.cell.2012.11.001 23217709

[B44] Iwasaki K. Ray P. D. Huang B. W. Sakamoto K. Kobayashi T. Tsuji Y. (2013). Role of AMP-activated protein kinase in ferritin H gene expression by resveratrol in human T cells. *Biochemistry* 52 5075–5083. 10.1021/bi400399f 23829535 PMC4108177

[B45] Jeon C. Y. Murray M. B. (2008). Diabetes mellitus increases the risk of active tuberculosis: a systematic review of 13 observational studies. *PLoS Med.* 5:e152. 10.1371/journal.pmed.0050152 18630984 PMC2459204

[B46] Jha A. K. Huang S. C. Sergushichev A. Lampropoulou V. Ivanova Y. Loginicheva E. (2015). Network integration of parallel metabolic and transcriptional data reveals metabolic modules that regulate macrophage polarization. *Immunity* 42 419–430. 10.1016/j.immuni.2015.02.005 25786174

[B47] Jia J. Abudu Y. P. Claude-Taupin A. Gu Y. Kumar S. Choi S. W. (2018). Galectins control mTOR in response to endomembrane damage. *Mol. Cell* 70 120.e8–135.e8. 10.1016/j.molcel.2018.03.009 29625033 PMC5911935

[B48] Kelly B. O’Neill L. A. (2015). Metabolic reprogramming in macrophages and dendritic cells in innate immunity. *Cell Res.* 25 771–784. 10.1038/cr.2015.68 26045163 PMC4493277

[B49] Kim J. Kundu M. Viollet B. Guan K. L. (2011). AMPK and mTOR regulate autophagy through direct phosphorylation of Ulk1. *Nat. Cell Biol.* 13 132–141. 10.1038/ncb2152 21258367 PMC3987946

[B50] Kim J. K. Lee H. M. Park K. S. Shin D. M. Kim T. S. Kim Y. S. (2017). MIR144^∗^ inhibits antimicrobial responses against *Mycobacterium tuberculosis* in human monocytes and macrophages by targeting the autophagy protein DRAM2. *Autophagy* 13 423–441. 10.1080/15548627.2016.1241922 27764573 PMC5324854

[B51] Kim T. S. Shin Y. H. Lee H. M. Kim J. K. Choe J. H. Jang J. C. (2017). Ohmyungsamycins promote antimicrobial responses through autophagy activation via AMP-activated protein kinase pathway. *Sci. Rep.* 7:3431. 10.1038/s41598-017-03477-3 28611371 PMC5469788

[B52] Kim Y. S. Lee H. M. Kim J. K. Yang C. S. Kim T. S. Jung M. (2017). PPAR-alpha activation mediates innate host defense through induction of tfeb and lipid catabolism. *J. Immunol.* 198 3283–3295. 10.4049/jimmunol.1601920 28275133

[B53] Kim S. Y. Yang C. S. Lee H. M. Kim J. K. Kim Y. S. Kim Y. R. (2018). ESRRA (estrogen-related receptor alpha) is a key coordinator of transcriptional and post-translational activation of autophagy to promote innate host defense. *Autophagy* 14 152–168. 10.1080/15548627.2017.1339001 28841353 PMC5846564

[B54] Koh Y. Lim H. Y. Ahn J. H. Lee J. L. Rha S. Y. Kim Y. J. (2013). Phase II trial of everolimus for the treatment of nonclear-cell renal cell carcinoma. *Ann. Oncol.* 24 1026–1031. 10.1093/annonc/mds582 23180114

[B55] Korolchuk V. I. Saiki S. Lichtenberg M. Siddiqi F. H. Roberts E. A. Imarisio S. (2011). Lysosomal positioning coordinates cellular nutrient responses. *Nat. Cell. Biol.* 13 453–460. 10.1038/ncb2204 21394080 PMC3071334

[B56] Krawczyk C. M. Holowka T. Sun J. Blagih J. Amiel E. DeBerardinis R. J. (2010). Toll-like receptor-induced changes in glycolytic metabolism regulate dendritic cell activation. *Blood* 115 4742–4749. 10.1182/blood-2009-10-249540 20351312 PMC2890190

[B57] Lachmandas E. Beigier-Bompadre M. Cheng S. C. Kumar V. van Laarhoven A. Wang X. (2016). Rewiring cellular metabolism via the AKT/mTOR pathway contributes to host defence against *Mycobacterium tuberculosis* in human and murine cells. *Eur. J. Immunol.* 46 2574–2586. 10.1002/eji.201546259 27624090 PMC5129526

[B58] Lee K. A. Cho K. C. Kim B. Jang I. H. Nam K. Kwon Y. E. (2018). Inflammation-modulated metabolic reprogramming is required for duox-dependent gut immunity in drosophila. *Cell Host Microbe* 23 338.e5–352.e5. 10.1016/j.chom.2018.01.011 29503179

[B59] Levine B. Deretic V. (2007). Unveiling the roles of autophagy in innate and adaptive immunity. *Nat. Rev. Immunol.* 7 767–777. 10.1038/nri2161 17767194 PMC7097190

[B60] Liu C. H. Liu H. Ge B. (2017). Innate immunity in tuberculosis: host defense vs pathogen evasion. *Cell Mol. Immunol.* 14 963–975. 10.1038/cmi.2017.88 28890547 PMC5719146

[B61] Lugo-Villarino G. Troegeler A. Balboa L. Lastrucci C. Duval C. Mercier I. (2018). The C-type lectin receptor DC-SIGN has an anti-inflammatory role in human M(IL-4) macrophages in response to *Mycobacterium tuberculosis*. *Front. Immunol.* 9:1123. 10.3389/fimmu.2018.01123 29946317 PMC6006465

[B62] MacMicking J. D. Taylor G. A. McKinney J. D. (2003). Immune control of tuberculosis by IFN-gamma-inducible LRG-47. *Science* 302 654–659. 10.1126/science.1088063 14576437

[B63] Madhavi Y. V. Gaikwad N. Yerra V. G. Kalvala A. K. Nanduri S. Kumar A. (2018). Targeting AMPK in diabetes and diabetic complications: energy homeostasis, autophagy and mitochondrial health. *Curr. Med. Chem.* 10.2174/0929867325666180406120051 [Epub ahead of print]. 29623826

[B64] Mahon R. N. Hafner R. (2015). Immune cell regulatory pathways unexplored as host-directed therapeutic targets for *Mycobacterium tuberculosis*: an opportunity to apply precision medicine innovations to infectious diseases. *Clin. Infect. Dis.* 61(Suppl. 3), S200–S216. 10.1093/cid/civ621 26409283 PMC4583576

[B65] Man S. M. Kanneganti T. D. (2016). Regulation of lysosomal dynamics and autophagy by CTSB/cathepsin B. *Autophagy* 12 2504–2505. 10.1080/15548627.2016.1239679 27786577 PMC5173259

[B66] Marcelo K. L. Means A. R. York B. (2016). The Ca(2+)/Calmodulin/CaMKK2 axis: nature’s metabolic CaMshaft. *Trends Endocrinol. Metab.* 27 706–718. 10.1016/j.tem.2016.06.001 27449752 PMC5035586

[B67] Marcinko K. Steinberg G. R. (2014). The role of AMPK in controlling metabolism and mitochondrial biogenesis during exercise. *Exp. Physiol.* 99 1581–1585. 10.1113/expphysiol.2014.082255 25261498

[B68] Martina J. A. Chen Y. Gucek M. Puertollano R. (2012). MTORC1 functions as a transcriptional regulator of autophagy by preventing nuclear transport of TFEB. *Autophagy* 8 903–914. 10.4161/auto.19653 22576015 PMC3427256

[B69] Matta S. K. Kumar D. (2016). Hypoxia and classical activation limits *Mycobacterium tuberculosis* survival by Akt-dependent glycolytic shift in macrophages. *Cell Death Discov.* 2:16022. 10.1038/cddiscovery.2016.22 27551515 PMC4979487

[B70] Mercer C. A. Kaliappan A. Dennis P. B. (2009). A novel, human Atg13 binding protein, Atg101, interacts with ULK1 and is essential for macroautophagy. *Autophagy* 5 649–662. 10.4161/auto.5.5.8249 19287211

[B71] Michelucci A. Cordes T. Ghelfi J. Pailot A. Reiling N. Goldmann O. (2013). Immune-responsive gene 1 protein links metabolism to immunity by catalyzing itaconic acid production. *Proc. Natl. Acad. Sci. U.S.A.* 110 7820–7825. 10.1073/pnas.1218599110 23610393 PMC3651434

[B72] Mizushima N. Levine B. Cuervo A. M. Klionsky D. J. (2008). Autophagy fights disease through cellular self-digestion. *Nature* 451 1069–1075. 10.1038/nature06639 18305538 PMC2670399

[B73] Moreira D. Silvestre R. Cordeiro-da-Silva A. Estaquier J. Foretz M. Viollet B. (2016). AMP-activated protein kinase as a target for pathogens: friends or foes? *Curr. Drug Targets* 17 942–953. 10.2174/1389450116666150416120559 25882224 PMC5387108

[B74] Neumann D. (2018). Is TAK1 a direct upstream kinase of AMPK? *Int. J. Mol. Sci.* 19:E2412. 10.3390/ijms19082412 30111748 PMC6121279

[B75] Ni Cheallaigh C. Keane J. Lavelle E. C. Hope J. C. Harris J. (2011). Autophagy in the immune response to tuberculosis: clinical perspectives. *Clin. Exp. Immunol.* 164 291–300. 10.1111/j.1365-2249.2011.04381.x 21438870 PMC3087924

[B76] Novikova D. S. Garabadzhiu A. V. Melino G. Barlev N. A. Tribulovich V. G. (2015). AMP-activated protein kinase: structure, function, and role in pathological processes. *Biochemistry* 80 127–144. 10.1134/S0006297915020017 25756529

[B77] O’Neill L. A. Hardie D. G. (2013). Metabolism of inflammation limited by AMPK and pseudo-starvation. *Nature* 493 346–355. 10.1038/nature11862 23325217

[B78] O’Neill L. A. Kishton R. J. Rathmell J. (2016). A guide to immunometabolism for immunologists. *Nat. Rev. Immunol.* 16 553–565. 10.1038/nri.2016.70 27396447 PMC5001910

[B79] O’Neill L. A. Pearce E. J. (2016). Immunometabolism governs dendritic cell and macrophage function. *J. Exp. Med.* 213 15–23. 10.1084/jem.20151570 26694970 PMC4710204

[B80] Ong C. W. Elkington P. T. Brilha S. Ugarte-Gil C. Tome-Esteban M. T. Tezera L. B. (2015). Neutrophil-derived MMP-8 drives AMPK-dependent matrix destruction in human pulmonary tuberculosis. *PLoS Pathog.* 11:e1004917. 10.1371/journal.ppat.1004917 25996154 PMC4440706

[B81] Ouimet M. Koster S. Sakowski E. Ramkhelawon B. van Solingen C. Oldebeken S. (2016). *Mycobacterium tuberculosis* induces the miR-33 locus to reprogram autophagy and host lipid metabolism. *Nat. Immunol.* 17 677–686. 10.1038/ni.3434 27089382 PMC4873392

[B82] Paik S. Kim J. K. Chung C. Jo E. K. (2018). Autophagy: a new strategy for host-directed therapy of tuberculosis. *Virulence* 10.1080/21505594.2018.1536598 [Epub ahead of print]. 30322337 PMC6550549

[B83] Pascual J. Royuela A. Fernandez A. M. Herrero I. Delgado J. F. Sole A. (2016). Role of mTOR inhibitors for the control of viral infection in solid organ transplant recipients. *Transpl. Infect. Dis.* 18 819–831. 10.1111/tid.12601 27600985

[B84] Peixoto C. A. Oliveira W. H. Araujo S. Nunes A. K. S. (2017). AMPK activation: role in the signaling pathways of neuroinflammation and neurodegeneration. *Exp. Neurol.* 298(Pt A), 31–41. 10.1016/j.expneurol.2017.08.013 28844606

[B85] Pineros A. R. Campos L. W. Fonseca D. M. Bertolini T. B. Gembre A. F. Prado R. Q. (2017). M2 macrophages or IL-33 treatment attenuate ongoing *Mycobacterium tuberculosis* infection. *Sci. Rep.* 7:41240. 10.1038/srep41240 28128217 PMC5269597

[B86] Pohanka E. (2006). Conversion to everolimus in maintenance patients–current clinical strategies. *Nephrol. Dial. Transplant.* 21(Suppl. 3), iii24–iii29. 10.1093/ndt/gfl301 16815853

[B87] Prantner D. Perkins D. J. Vogel S. N. (2017). AMP-activated kinase (AMPK) promotes innate immunity and antiviral defense through modulation of stimulator of interferon genes (STING) signaling. *J. Biol. Chem.* 292 292–304. 10.1074/jbc.M116.763268 27879319 PMC5217687

[B88] Qi X. Man S. M. Malireddi R. K. Karki R. Lupfer C. Gurung P. (2016). Cathepsin B modulates lysosomal biogenesis and host defense against *Francisella novicida* infection. *J. Exp. Med.* 213 2081–2097. 10.1084/jem.20151938 27551156 PMC5030800

[B89] Reddy V. P. Chinta K. C. Saini V. Glasgow J. N. Hull T. D. Traylor A. (2018). Ferritin H deficiency in myeloid compartments dysregulates host energy metabolism and increases susceptibility to *Mycobacterium tuberculosis* infection. *Front. Immunol.* 9:860. 10.3389/fimmu.2018.00860 29774023 PMC5943674

[B90] Rekha R. S. Rao Muvva S. S. Wan M. Raqib R. Bergman P. Brighenti S. (2015). Phenylbutyrate induces LL-37-dependent autophagy and intracellular killing of *Mycobacterium tuberculosis* in human macrophages. *Autophagy* 11 1688–1699. 10.1080/15548627.2015.1075110 26218841 PMC4590658

[B91] Riffelmacher T. Richter F. C. Simon A. K. (2018). Autophagy dictates metabolism and differentiation of inflammatory immune cells. *Autophagy* 14 199–206. 10.1080/15548627.2017.1362525 28806133 PMC5902226

[B92] Roczniak-Ferguson A. Petit C. S. Froehlich F. Qian S. Ky J. Angarola B. (2012). The transcription factor TFEB links mTORC1 signaling to transcriptional control of lysosome homeostasis. *Sci. Signal.* 5:ra42. 10.1126/scisignal.2002790 22692423 PMC3437338

[B93] Ruderman N. B. Carling D. Prentki M. Cacicedo J. M. (2013). AMPK, insulin resistance, and the metabolic syndrome. *J. Clin. Invest.* 123 2764–2772. 10.1172/JCI67227 23863634 PMC3696539

[B94] Sag D. Carling D. Stout R. D. Suttles J. (2008). Adenosine 5’-monophosphate-activated protein kinase promotes macrophage polarization to an anti-inflammatory functional phenotype. *J. Immunol.* 181 8633–8641. 10.4049/jimmunol.181.12.8633 19050283 PMC2756051

[B95] Sahu S. K. Kumar M. Chakraborty S. Banerjee S. K. Kumar R. Gupta P. (2017). MicroRNA 26a (miR-26a)/KLF4 and CREB-C/EBPbeta regulate innate immune signaling, the polarization of macrophages and the trafficking of *Mycobacterium tuberculosis* to lysosomes during infection. *PLoS Pathog.* 13:e1006410. 10.1371/journal.ppat.1006410 28558034 PMC5466338

[B96] Schiebler M. Brown K. Hegyi K. Newton S. M. Renna M. Hepburn L. (2015). Functional drug screening reveals anticonvulsants as enhancers of mTOR-independent autophagic killing of *Mycobacterium tuberculosis* through inositol depletion. *EMBO Mol. Med.* 7 127–139. 10.15252/emmm.201404137 25535254 PMC4328644

[B97] Schorey J. S. Schlesinger L. S. (2016). Innate immune responses to tuberculosis. *Microbiol Spectr* 4 3–31. 10.1128/microbiolspec.TBTB2-0010-2016 28087945

[B98] Settembre C. Di Malta C. Polito V. A. Garcia Arencibia M. Vetrini F. Erdin S. (2011). TFEB links autophagy to lysosomal biogenesis. *Science* 332 1429–1433. 10.1126/science.1204592 21617040 PMC3638014

[B99] Shah S. Z. A. Zhao D. Hussain T. Yang L. (2017). Role of the AMPK pathway in promoting autophagic flux via modulating mitochondrial dynamics in neurodegenerative diseases: Insight into prion diseases. *Ageing Res. Rev.* 40 51–63. 10.1016/j.arr.2017.09.004 28903070

[B100] Shi L. Eugenin E. A. Subbian S. (2016). Immunometabolism in tuberculosis. *Front. Immunol.* 7:150. 10.3389/fimmu.2016.00150 27148269 PMC4838633

[B101] Shi L. Z. Wang R. Huang G. Vogel P. Neale G. Green D. R. (2011). HIF1alpha-dependent glycolytic pathway orchestrates a metabolic checkpoint for the differentiation of TH17 and Treg cells. *J. Exp. Med.* 208 1367–1376. 10.1084/jem.20110278 21708926 PMC3135370

[B102] Shin D. M. Yuk J. M. Lee H. M. Lee S. H. Son J. W. Harding C. V. (2010). Mycobacterial lipoprotein activates autophagy via TLR2/1/CD14 and a functional vitamin D receptor signalling. *Cell Microbiol.* 12 1648–1665. 10.1111/j.1462-5822.2010.01497.x 20560977 PMC2970753

[B103] Silwal P. Kim J. K. Yuk J. M. Jo E. K. (2018). AMP-Activated protein kinase and host defense against infection. *Int. J. Mol. Sci.* 19:E3495. 10.3390/ijms19113495 30404221 PMC6274990

[B104] Singh P. Subbian S. (2018). Harnessing the mTOR pathway for tuberculosis treatment. *Front. Microbiol.* 9:70. 10.3389/fmicb.2018.00070 29441052 PMC5797605

[B105] Singh S. B. Davis A. S. Taylor G. A. Deretic V. (2006). Human IRGM induces autophagy to eliminate intracellular mycobacteria. *Science* 313 1438–1441. 10.1126/science.1129577 16888103

[B106] Singh S. B. Ornatowski W. Vergne I. Naylor J. Delgado M. Roberts E. (2010). Human IRGM regulates autophagy and cell-autonomous immunity functions through mitochondria. *Nat. Cell Biol.* 12 1154–1165. 10.1038/ncb2119 21102437 PMC2996476

[B107] Singhal A. Jie L. Kumar P. Hong G. S. Leow M. K. Paleja B. (2014). Metformin as adjunct antituberculosis therapy. *Sci. Transl. Med.* 6:263ra159. 10.1126/scitranslmed.3009885 25411472

[B108] Stutz M. D. Clark M. P. Doerflinger M. Pellegrini M. (2018). *Mycobacterium tuberculosis*: rewiring host cell signaling to promote infection. *J. Leukoc. Biol.* 103 259–268. 10.1002/JLB.4MR0717-277R 29345343 PMC6446910

[B109] Sun J. Mu Y. Jiang Y. Song R. Yi J. Zhou J. (2018). Inhibition of p70 S6 kinase activity by A77 1726 induces autophagy and enhances the degradation of superoxide dismutase 1 (SOD1) protein aggregates. *Cell Death Dis.* 9:407. 10.1038/s41419-018-0441-0 29540819 PMC5851998

[B110] Szrejder M. Piwkowska A. (2019). AMPK signaling: implications for podocyte biology in diabetic nephropathy. *Biol. Cell* 10.1111/boc.201800077 [Epub ahead of print]. 30702162

[B111] Tang B. L. (2016). Sirt1 and the mitochondria. *Mol. Cells* 39 87–95. 10.14348/molcells.2016.2318 26831453 PMC4757807

[B112] Tannahill G. M. Curtis A. M. Adamik J. Palsson-McDermott E. M. McGettrick A. F. Goel G. (2013). Succinate is an inflammatory signal that induces IL-1beta through HIF-1alpha. *Nature* 496 238–242. 10.1038/nature11986 23535595 PMC4031686

[B113] Tiwari S. Choi H. P. Matsuzawa T. Pypaert M. MacMicking J. D. (2009). Targeting of the GTPase Irgm1 to the phagosomal membrane via PtdIns(3,4)P(2) and PtdIns(3,4,5)P(3) promotes immunity to mycobacteria. *Nat. Immunol.* 10 907–917. 10.1038/ni.1759 19620982 PMC2715447

[B114] Wang G. S. Hoyte C. (2018). Review of biguanide (metformin) toxicity. *J. Inten. Care Med.* 10.1177/0885066618793385 [Epub ahead of print]. 30126348

[B115] Watson R. O. Manzanillo P. S. Cox J. S. (2012). Extracellular *M. tuberculosis* DNA targets bacteria for autophagy by activating the host DNA-sensing pathway. *Cell* 150 803–815. 10.1016/j.cell.2012.06.040 22901810 PMC3708656

[B116] WHO (2017). *Global Tuberculosis Report 2017.* Geneva: World Health Organization.

[B117] Williams N. C. O’Neill L. A. J. (2018). A role for the krebs cycle intermediate citrate in metabolic reprogramming in innate immunity and inflammation. *Front. Immunol.* 9:141. 10.3389/fimmu.2018.00141 29459863 PMC5807345

[B118] Wong P. M. Puente C. Ganley I. G. Jiang X. (2013). The ULK1 complex: sensing nutrient signals for autophagy activation. *Autophagy* 9 124–137. 10.4161/auto.23323 23295650 PMC3552878

[B119] Wu S. B. Wu Y. T. Wu T. P. Wei Y. H. (2014). Role of AMPK-mediated adaptive responses in human cells with mitochondrial dysfunction to oxidative stress. *Biochim. Biophys. Acta* 1840 1331–1344. 10.1016/j.bbagen.2013.10.034 24513455

[B120] Xie M. Zhang D. Dyck J. R. Li Y. Zhang H. Morishima M. (2006). A pivotal role for endogenous TGF-beta-activated kinase-1 in the LKB1/AMP-activated protein kinase energy-sensor pathway. *Proc. Natl. Acad. Sci. U.S.A.* 103 17378–17383. 10.1073/pnas.0604708103 17085580 PMC1859937

[B121] Xu X. Sun J. Song R. Doscas M. E. Williamson A. J. Zhou J. (2017). Inhibition of p70 S6 kinase (S6K1) activity by A77 1726, the active metabolite of leflunomide, induces autophagy through TAK1-mediated AMPK and JNK activation. *Oncotarget* 8 30438–30454. 10.18632/oncotarget.16737 28389629 PMC5444754

[B122] Yang C. S. Kim J. J. Lee H. M. Jin H. S. Lee S. H. Park J. H. (2014). The AMPK-PPARGC1A pathway is required for antimicrobial host defense through activation of autophagy. *Autophagy* 10 785–802. 10.4161/auto.28072 24598403 PMC5119058

[B123] Yang C. S. Song C. H. Lee J. S. Jung S. B. Oh J. H. Park J. (2006). Intracellular network of phosphatidylinositol 3-kinase, mammalian target of the rapamycin/70 kDa ribosomal S6 kinase 1, and mitogen-activated protein kinases pathways for regulating mycobacteria-induced IL-23 expression in human macrophages. *Cell Microbiol.* 8 1158–1171. 10.1111/j.1462-5822.2006.00699.x 16819968

[B124] Yao F. Zhang M. Chen L. (2016). 5’-Monophosphate-activated protein kinase (AMPK) improves autophagic activity in diabetes and diabetic complications. *Acta Pharm. Sin. B* 6 20–25. 10.1016/j.apsb.2015.07.009 26904395 PMC4724658

[B125] Yorke E. Atiase Y. Akpalu J. Sarfo-Kantanka O. Boima V. Dey I. D. (2017). The bidirectional relationship between tuberculosis and diabetes. *Tuberc. Res. Treat.* 2017:1702578. 10.1155/2017/1702578 29270319 PMC5705893

[B126] Yuk J. M. Kim T. S. Kim S. Y. Lee H. M. Han J. Dufour C. R. (2015). Orphan nuclear receptor ERRalpha controls macrophage metabolic signaling and A20 expression to negatively regulate tlr-induced inflammation. *Immunity* 43 80–91. 10.1016/j.immuni.2015.07.003 26200012

[B127] Yuk J. M. Shin D. M. Lee H. M. Yang C. S. Jin H. S. Kim K. K. (2009). Vitamin D3 induces autophagy in human monocytes/macrophages via cathelicidin. *Cell Host Microbe* 6 231–243. 10.1016/j.chom.2009.08.004 19748465

[B128] Zhang D. Wang W. Sun X. Xu D. Wang C. Zhang Q. (2016). AMPK regulates autophagy by phosphorylating BECN1 at threonine 388. *Autophagy* 12 1447–1459. 10.1080/15548627.2016.1185576 27304906 PMC5082788

[B129] Zhang J. Wang Y. Liu X. Dagda R. K. Zhang Y. (2017). How AMPK and PKA interplay to regulate mitochondrial function and survival in models of ischemia and diabetes. *Oxid. Med. Cell Longev.* 2017:4353510. 10.1155/2017/4353510 29391924 PMC5748092

[B130] Zhu J. Wang K. Z. Chu C. T. (2013). After the banquet: mitochondrial biogenesis, mitophagy, and cell survival. *Autophagy* 9 1663–1676. 10.4161/auto.24135 23787782 PMC4028332

